# An Integrated Therapeutic Strategy for Cardiorenal Syndrome to Target Oxidative Stress, Inflammation, and Fibrosis

**DOI:** 10.31083/RCM50347

**Published:** 2026-08-18

**Authors:** Xinwei Wang, Qiongyao He, Desen Yang, Yaoyao Guo, Siyu Fu, Wu He

**Affiliations:** ^1^College of Pharmacy, Hubei University of Chinese Medicine, 430065 Wuhan, Hubei, China; ^2^Division of Endocrinology, Department of Internal Medicine, Tongji Hospital, Tongji Medical College, Huazhong University of Science and Technology, 430030 Wuhan, Hubei, China; ^3^Hubei Shizhen Laboratory, 430060 Wuhan, Hubei, China; ^4^Chinese Materia Medica Processing Engineering Center of Hubei Province, Hubei University of Chinese Medicine, 430065 Wuhan, Hubei, China; ^5^Division of Cardiology, Departments of Internal Medicine, Tongji Hospital, Tongji Medical College, Huazhong University of Science and Technology, 430030 Wuhan, Hubei, China; ^6^Hubei Key Laboratory of Genetics and Molecular Mechanisms of Cardiological Disorders, 430030 Wuhan, Hubei, China

**Keywords:** cardiorenal syndrome, oxidative stress, inflammation, fibrosis, pathophysiology, combined therapy

## Abstract

Cardiorenal syndrome (CRS) is characterized by a complex, bidirectional interaction between cardiac and renal dysfunction, posing a significant challenge in clinical practice. Although these two organ dysfunctions share common pathophysiological pathways, there remains a major unmet need for therapies that specifically target these shared mechanisms. Oxidative stress, chronic low-grade inflammation, and progressive tissue fibrosis are pivotal, interconnected drivers involved in the progression of both chronic heart failure and chronic kidney disease, constituting a critical pathological triad in CRS. This review aims to systematically elucidate the interplay among reactive oxygen species (ROS), immune cell infiltration, proinflammatory cytokine signaling, and fibrotic pathway activation in the pathogenesis of CRS. We also provide a comprehensive overview of the current landscape of novel pharmacological candidates that target key nodes in the oxidative stress–inflammation–fibrosis axis, including small molecules, biologics, and repurposed drugs. By integrating preclinical evidence with clinical trial data, this review highlights the therapeutic potential of concurrently attenuating oxidative stress, inflammation, and fibrosis to disrupt the vicious cycle of CRS and ultimately improve patient outcomes.

## 1. Introduction to Cardiorenal Syndrome

Cardiorenal syndrome (CRS) is a complex condition that entails the bidirectional dysfunction of the heart and the kidneys, with the deterioration of one organ contributing to the dysfunction of the other [1]. As of 2019, an estimated 700 million people worldwide were living with chronic kidney disease (CKD) [2], while 523 million suffer from cardiovascular disease [3]. In a review conducted in 2019, the overall prevalence of co-existing chronic kidney disease in the heart failure population was reported to be 49% [4]. A cross-sectional study of the National Health and Nutrition Examination Survey (NHANES) dataset in the United States from 1999 to 2020 revealed that 25% of the study subjects had at least one form of heart, kidney, or metabolic disease [5]. Clinical studies have further revealed that as of 2017, the number of adult heart failure patients worldwide had reached approximately 64.3 million [6]. Among this patient population, about one-third have an estimated glomerular filtration rate (eGFR) of 60 mL/min/1.73 m^2^ or less [7]. This cardiac and renal comorbidity is significantly associated with a higher risk of adverse prognosis, as the incidence of mortality in heart failure patients with concomitant renal dysfunction is estimated at 57.8%–73.3%, significantly higher than that in heart failure patients without concomitant kidney injury (40.4%) [8,9]. The close interplay between obesity, type 2 diabetes mellitus (T2DM), cardiovascular disease (CVD), and CKD has become increasingly prevalent.

Recognizing the extensive interplay among cardiovascular, kidney, and metabolic diseases, the American Heart Association (AHA) has recently introduced the umbrella term cardiovascular-kidney-metabolic (CKM) syndrome, a broader conceptual framework distinct from, though related to, the traditional classification of CRS [10]. The five classifications of CRS and their definitions are as follows: CRS type 1 (acute cardiorenal syndrome) is defined as the acute worsening of cardiac function leading to acute kidney injury (AKI); CRS type 2 (chronic cardiorenal syndrome) refers to chronic heart failure leading to progressive and persistent CKD; CRS type 3 (acute renocardiac syndrome) entails acute kidney injury causing acute cardiac dysfunction; CRS type 4 (chronic renocardiac syndrome) involves CKD leading to progressive chronic heart failure; and CRS type 5 (secondary CRS) is cardiorenal dysfunction caused by a third common disease (such as sepsis, diabetes mellitus, or systemic lupus erythematosus) [11]. The chronic CRS subtypes (Type 2 and Type 4) are clinically important entities due to their high prevalence and complex management, and they are strongly associated with poor long-term outcomes, including increased risks of cardiovascular mortality and progression to end-stage renal disease [12].

Animal models are often used to reproduce key pathological features of human CRS in an experimental context. Corresponding to the clinical subtypes of CRS, coronary artery ligation (CL) and aortic fistula (ACF) models are commonly used to simulate Type 1 and Type 2 disease, respectively [13,14]. In the CL model, coronary artery ligation is used to induce myocardial infarction, which in turn leads to heart failure. The ACF model, in contrast, involves surgically establishing a shunt between the abdominal aorta and inferior vena cava. For the kidney-to-heart direction, Surgical nephrectomy (SNX) is the most widely used approach for simulating Types 3 and 4 CRS [15]. The two main approaches to SNX modeling involve either unilateral nephrectomy followed one week later by the removal of the pole of the remaining kidney, or the ligation of two of the three renal arteries before contralateral nephrectomy. While other models exist for studying specific aspects of CRS pathophysiology, these representative approaches have been used to validate the clinical observation that kidney injury can directly lead to cardiac disease.

Recent research advances have progressively moved away from rigid distinctions between the five clinical subtypes of CRS, advocating instead for a unifying pathophysiological framework centered on the interconnected pathways of oxidative stress, inflammation, and fibrosis [1,16]. The links between these pathways constitute a pathological triad that prompts the development of CRS independent of the precipitating organ [17]. In addition to being a marker of the progression of CRS, fibrosis is also considered a key pathogenic driver of this disease [18]. Oxidative stress and inflammation, as the main initiating and mediating factors involved in the fibrotic process, promote extracellular matrix deposition by activating transforming growth factor-β1 (TGF-β1)/small mothers against decapentaplegic (Smad) and other pro-fibrotic signaling pathways, leading to tissue remodeling and dysfunction, forming a vicious self-reinforcing cycle. In addition, recently identified mechanisms such as intestinal flora imbalance and abnormal non-coding RNA regulation have also been found to be involved in this pathological network, further enriching the understanding of the molecular basis of CRS [19].

The prevalence of CRS is gradually increasing, especially among adults and patients with comorbidities such as hypertension, diabetes mellitus, and obesity, which can induce and exacerbate both cardiac and renal dysfunction [16,20]. Metabolic abnormalities further impair cardiac and renal function and accelerate the progression of CRS through various pathways, including hemodynamic changes, excessive neuroendocrine system activation, endothelial dysfunction, and the secretion of proinflammatory factors by adipose tissue [21]. This dysfunction of adipose tissue is believed to play a central role in obesity-related CRS. In particular, excessive free fatty acids released by adipose tissue can directly induce oxidative stress and inflammation, thereby establishing the pathological foundation for the development of cardiorenal-metabolic syndrome [21]. Efforts to fully characterize the molecular crosstalk between these pathways may therefore inform the development of rational combinations of therapeutic approaches capable of addressing more than one aspect of CRS pathogenesis [22,23].

## 2. Oxidative Stress in Cardiorenal Syndrome

### 2.1 Sources and Mechanisms of Oxidative Stress

Oxidative stress is a common pathological feature of CRS and other conditions such as chronic kidney disease and heart failure that results from an imbalance between ROS production and antioxidant defense system activity [24,25,26]. Key endogenous sources of ROS include mitochondrial ROS production, as well as several enzyme-mediated pathways involving NADPH oxidases (NOX), uncoupled endothelial nitric oxide synthase (eNOS), xanthine oxidase (XO), cytochrome P450 (CYP450), and lipoxygenase activity [27]. Multiple enzymatic systems contribute to reactive oxygen species (ROS) generation in cardiac and renal tissues, with NOX playing a particularly significant role [28]. These NOX family enzymes are key sources of ROS generation in CRS. Notably, NOX1, NOX2, and NOX4 exhibit significantly elevated expression in heart and kidney tissues, with their expression levels showing a positive correlation with the severity of cardiac and renal injury [29,30,31]. Consistently, the inhibition of NOX activity can effectively reduce oxidative damage, inflammatory infiltration, and fibrosis. As another ROS source, XO activity is significantly negatively correlated with left ventricular ejection fraction, and positively correlated with brain natriuretic peptide, urea nitrogen, and proteinuria levels [30].

Mitochondrial dysfunction constitutes another central driver of oxidative stress in CRS [32,33]. In the context of Type 4 CRS, impaired mitochondrial electron transport chain activity is evident in cardiac and renal tissues, resulting in reduced ATP synthesis and abnormal ROS release [34]. This mitochondrial homeostatic imbalance further leads to disturbances in mitochondrial dynamics such as excessive fission, impaired mitophagy, and bioenergetic failure, forming a deleterious feedback cycle [35]. Experimental evidence has shown that mitochondrial oxygen consumption in the myocardium and kidney is significantly reduced in CRS models, accompanied by cytochrome c release and the activation of apoptotic pathways [31]. Notably, NOX and mitochondrial activity are not siloed from one another such that they interact significantly: NOX-derived ROS can damage mitochondrial membrane potential, while mitochondrial dysfunction can activate NOX, forming a positive ROS-induced ROS release feedback loop [36]. In addition, other oxidative pathways such as eNOS uncoupling and XO activation are also synergistically involved in this process [37,38]. Given the pivotal position of oxidative stress in the pathogenesis of CRS, targeting NOX inhibition, mitochondrial protection, or enhancing endogenous antioxidant capacity have emerged as candidate therapeutic strategies [39].

### 2.2 Consequences of Increased Oxidative Stress in Heart and Kidney

Oxidative stress constitutes a central pathophysiological mechanism underpinning the reciprocal dysfunction characteristic of CRS [40], as it simultaneously impairs both cardiac and renal function through overlapping and distinct pathological pathways. In cardiac tissue, the overproduction of ROS directly instigates cardiomyocyte apoptosis, pathological hypertrophy, and adverse electrical remodeling [41,42,43]. Notably, this cardiac oxidative injury is further exacerbated by renal dysfunction in CRS, as uremic toxins accumulate and suppress cardiac antioxidant defenses, creating a vicious cycle between the two organs [44]. Within the renal compartment, oxidative injury manifests as podocyte depletion, glomerulosclerosis, and tubular-interstitial fibrosis, driven by lipid peroxidation, protein carbonylation, and mitochondrial DNA damage [45]. Similarly, cardiac-derived pro-oxidative factors, such as angiotensin II and pro-inflammatory cytokines, can diffuse systemically to amplify renal oxidative stress, reinforcing the reciprocal nature of CRS [46].

The amplification of oxidative stress in CRS is perpetuated by a confluence of upstream metabolic and pathological drivers. Persistent hyperglycemia, a prevalent comorbidity in CRS, contributes to the multifaceted disruption of redox homeostasis by activating the polyol and hexosamine pathways, promoting the formation of advanced glycation end products (AGEs), and stimulating protein kinase C (PKC) signaling [45]. These pathways enhance mitochondrial ROS generation and suppress endogenous antioxidant defenses by inhibiting the Nrf2/Kelch-like erythroid cell-derived protein with CNC homology (ECH)-associated protein 1 (Keap1)/ARE transcriptional program [47]. In cardiac tissue, hyperglycemia-induced oxidative stress disrupts calcium handling in cardiomyocytes and promotes cardiac fibroblast activation, accelerating cardiac hypertrophy and fibrosis [48]. Concurrently, the accumulation of advanced oxidation protein products (AOPPs) in CKD directly inflicts podocyte injury and drives the progression of focal segmental glomerulosclerosis [49]. Thus, the uremic milieu itself becomes a self-perpetuating source of oxidative burden.

At the molecular level, ROS function as critical second messengers that bridge oxidative stress to sterile inflammation and fibrogenesis. Specifically, elevated ROS levels trigger the phosphorylation and subsequent proteasomal degradation of the inhibitor of κB (IκB) via the activation of the IκB kinase (IKK) complex. This liberates nuclear Factor kappa-B (NF-κB), which subsequently translocates into the nucleus, where it initiates the transcription of pro-inflammatory cytokines such as tumor necrosis factor-α (TNF-α) and interleukin-6 (IL-6) [50]. In parallel, oxidative stress disrupts the inhibitory binding between thioredoxin and thioredoxin-interacting protein (TXNIP). The liberated TXNIP then directly engages the NOD-like receptor family pyrin domain-containing protein 3 (NLRP3) inflammasome scaffold, facilitating caspase-1 activation and the maturation of interleukin-1β (IL-1β) [51,52]. NF-κB and the NLRP3 inflammasome serve as core regulators of the inflammatory response such that the synergistic activation of the two can trigger a sustained inflammatory response, causing irreversible damage to the heart and kidneys. They also establish a mutually reinforcing loop with oxidative stress, accelerating the process of cardiac and renal fibrosis.

In addition to these canonical inflammatory and fibrotic pathways, recent evidence implicates the synergistic interplay between oxidative stress and regulated cell death modalities, particularly ferroptosis, which contributes to both cardiac and renal injury in CRS. Hyperglycemia-induced oxidative stress has been shown to downregulate glutathione peroxidase 4 (GPX4), a key phospholipid peroxidase, leading to the accumulation of lipid peroxidation products, including malondialdehyde (MDA) and 4-hydroxynonenal (4-HNE) in experimental diabetic nephropathy [53]. This suggests that oxidative stress not only initiates inflammatory signaling but also promotes ferroptosis in kidney cells, exacerbating tubular damage. In cardiac tissue, oxidative stress similarly induces ferroptosis: downregulation of GPX4 by ROS leads to lipid peroxidation in cardiomyocytes, resulting in cardiomyocyte loss, myocardial dysfunction, and increased susceptibility to arrhythmias [54]. This oxidative assault manifests with distinct pathological signatures in each organ. In the heart, ROS directly compromise contractile function by inducing cardiomyocyte apoptosis and promoting adverse hypertrophic remodeling, while also disrupting excitation-contraction coupling through oxidative modification of ion channels and calcium handling proteins [55]. In the kidney, the vulnerability of tubular epithelial cells to oxidative stress stems from their high mitochondrial density and metabolic activity; ROS trigger tubular cell death, activate pro-fibrotic signaling in interstitial fibroblasts, and drive podocyte injury, collectively leading to proteinuria and glomerulosclerosis [29]. In summary, oxidative stress serves as a critical signal integrator, translating metabolic disturbances (hyperglycemia, AOPPs) into downstream inflammatory and fibrotic programs via NF-κB/NLRP3 signaling, while simultaneously sensitizing both cardiac and renal cells to ferroptosis. This coordinated injury to both organs perpetuates the reciprocal dysfunction characteristic of CRS, making redox balance a key therapeutic target. Consequently, therapeutic strategies aimed at restoring redox balance represent a rational and promising approach to slowing CKD progression and mitigating the systemic deterioration seen in CRS. Such strategies include inhibiting ROS generation, bolstering Nrf2-mediated antioxidant defenses, or blocking upstream triggers such as AOPPs [56,57,58].

## 3. Inflammatory Pathways in Cardiorenal Syndrome

### 3.1 The Inflammatory-Fibrotic Axis in CRS

In CRS, acute or chronic cardiorenal injury leads to hyperactive inflammation and fibrotic remodeling, associated with injury-mediated immune cell infiltration and myofibroblast activation [16]. In addition, the transition from inflammation to fibrosis is closely related to the transition of macrophages from M1 to M2 phenotypes, which is accompanied by the induction of a stronger TGF-β response [59]. Long-term inflammatory reactions can disrupt hemodynamic pathways, contribute to dysregulated oxidative stress, and induce the production of cytokines and growth factors. These processes further initiate fibrosis-related stimuli that shape the pathological remodeling of heart and kidney tissues, leading to the deterioration of cardiac and renal function [17]. The inflammatory response lays the foundation for a pre-fibrotic microenvironment in the heart and kidney, and it further propels subsequent fibrotic remodeling through the action of activated myofibroblasts, which ultimately contributes to cardiorenal dysfunction [15]. For example, the Wnt/β-catenin pathway is co-activated in heart and kidney tissues, whereupon it can mediate cardiac hypertrophy and fibrosis, in addition to promoting renal fibrosis by inhibiting klotho expression and inducing renal tubular epithelial cell damage [60]. The TNF-like weak inducer of apoptosis/fibroblast growth factor-inducible molecule 14 (TWEAK/Fn14) signal axis has also been confirmed to drive inflammation, apoptosis, and fibrosis in cardiorenal tissues, establishing it as an important molecular hub connecting these dual forms of organ damage [61]. The formation of a fibrotic microenvironment depends on the establishment of a “fibrogenic niche” composed of abnormally deposited extracellular matrix, infiltrating immune cells, endothelial cells, and soluble factors. This fibrotic niche provides ideal conditions for the activation and proliferation of fibroblasts, while also impeding the processes of tissue repair and regeneration [62,63]. Notably, oxidative stress causes direct damage to the glomeruli and tubules in addition to promoting the release of inflammatory factors through NF-κB pathway activation, propagating an oxidative stress-inflammation-fibrosis feedback loop [64]. Preclinical studies have validated TGF-β, Wnt/β-catenin, and TWEAK/Fn14 as effective anti-fibrotic targets in CRS [60,65,66].

### 3.2 Key Inflammatory Mediators and Signaling Pathways

There are multiple important inflammatory mediators (including IL-1β, IL-6, TNF-α, High-mobility group box 1 [HMGB1]) and signaling pathways (NLRP3 inflammasome, NF-κB, TGF-β-activated kinase 1/Inhibitor of nuclear factor κB kinase β/Interferon regulatory factor 5 [TAK1-IKKβ-IRF5], Renin-angiotensin-aldosterone system [RAAS]) that are involved in the pathogenesis of CRS and which create an intricate inflammatory network that reinforces organ damage and fibrosis [22,23,67,68,69].

As a key molecular platform connecting cellular stress and severe inflammation, NLRP3 inflammasome activation strictly depends on the synergistic effects of dual signaling pathways [70]. An initial priming signal is provided by the activation of the NF-κB pathway triggered by damage-associated molecular patterns (DAMPs) or proinflammatory cytokines, leading to the significant upregulation of NLRP3 and precursor caspase-1. Subsequent ROS accumulation, mitochondrial dysfunction, or crystalline substance accumulation can then induce the assembly of the NLRP3 inflammasome complex [71,72]. This complex, in turn, mediates two key biological effects by catalyzing the cleavage of precursor caspase-1 to produce active caspase-1. Caspase-1 then activates gasdermin D (GSDMD) to initiate pyroptosis, a highly pro-inflammatory form of programmed cell death, in addition to processing IL-1β and IL-18 via proteolytic cleavage to produce mature secreted cytokines [73]. A variety of endogenous danger signals have also been confirmed to be involved in the regulation of the NLRP3 inflammasome. For example, oxidized mitochondrial DNA (mtDNA) released into the cytoplasm can engage multiple inflammatory pathways. While it can directly bind to and activate the NLRP3 inflammasome, it is also a potent activator of the cGAS-STING signaling axis, which has recently been shown to promote NLRP3 inflammasome activation through various mechanisms, such as inducing autophagy dysfunction [74,75]. These multi-level regulatory mechanisms jointly determine the threshold and intensity of inflammasome activation. Interventional strategies targeting the NLRP3 inflammasome pathway have shown significant therapeutic potential. In studies of atherosclerosis, Salvianolic acid B improves plaque stability by blocking the NF-κB/NLRP3 pathway [76]. More importantly, MCC950, a specific NLRP3 inhibitor, has shown anti-fibrotic effects in an animal model of a variety of fibrotic diseases [77]. Together, these pieces of evidence support targeting the NLRP3 inflammasome as a viable strategy for blocking the vicious cycle of inflammation and fibrosis.

As a representative DAMP, HMGB1 is a key inflammatory response mediator, and the signaling pathways it regulates also serve as a core link connecting cellular stress and the amplification of inflammation. During cellular stress, necrosis, or when actively secreted, HMGB1 is released into the extracellular environment and efficiently activates the NF-κB signaling pathway by specifically binding to the receptor for advanced glycation end products (RAGE) and Toll-like receptors 2 (TLR2) and 4 (TLR4) [78]. Sustained activation of this pathway drives transcriptional expression of a variety of proinflammatory cytokines, including TNF-α, IL-6, and monocyte chemoattractant protein-1 (MCP-1) [79]. These factors further amplify the inflammatory cascade and participate in tissue damage and fibrotic processes. This HMGB1/RAGE/TLR/NF-κB axis has been identified as an important amplifier in a variety of inflammatory diseases. For example, in a rat model of sepsis-induced acute kidney injury, the release of HMGB1 significantly upregulates the activity of NF-κB, leading to the massive production of inflammatory factors such as TNF-α, IL-6, and MCP-1, thereby promoting the infiltration of monocytes, macrophages, and neutrophils into target organs, exacerbating tissue inflammatory damage [80]. This vicious cycle is particularly prominent in the chronic inflammatory microenvironment, leading to the continuous exposure of tissues to oxidative stress and pro-inflammatory factors, eventually destroying the integrity of organ structure and function [81]. The HMGB1/RAGE/TLR/NF-κB axis also synergizes with the actions of the NLRP3 inflammasome, as activated NF-κB can directly upregulate the transcriptional expression of NLRP3, providing a basis for inflammasome activation and jointly promoting the vicious cycle of inflammation and fibrosis [80].

These interlinked mechanisms, including NLRP3-mediated pyroptosis, HMGB1/NF-κB-regulated cytokine storm, and fibroblast-intrinsic inflammatory signaling, comprise a multi-dimensional inflammatory engine underlying CRS pathogenesis. They show that initial injury, in the context of oxidative stress and DAMPs, is converted into a chronic cascading, self-sustaining form of inflammation that directly drives key fibrogenic effectors. This conceptual explanation highlights the potential feasibility of developing therapeutic interventions targeting numerous nodes synergistically to interrupt the inflammatory-fibrotic cycle that characterizes CRS.

RAAS overactivation represents another major inflammatory pathway in CRS [82]. Beyond its well-recognized role in mediating hemodynamic alterations, including increased systemic vascular resistance, sodium and water retention, and elevated cardiac afterload that directly burden both cardiac and renal function, RAAS activation exerts profound pro-inflammatory effects that perpetuate organ damage [15]. Notably, the pro-inflammatory actions of RAAS do not operate in isolation; instead, they engage in extensive crosstalk with other core pathological processes of CRS through damaging feedback processes [15]. Furthermore, uremic toxins that accumulate in CRS, such as indoxyl sulfate and p-cresol sulfate, have been shown to activate NF-κB, apoptosis signal-regulating kinase 1 (ASK1), and downstream mitogen-activated protein kinases, including p38 mitogen-activated protein kinases (p38-MAPK) and extracellular signal-related kinases (ERK1/2), further amplifying the inflammatory response [18,83]. This convergence of RAAS-mediated inflammation and uremic toxin-induced signaling cascades underscores the multifaceted nature of inflammatory dysregulation in CRS, highlighting the need for targeted interventions that address these interconnected pathways to mitigate disease progression.

### 3.3 Immune Cell-Mediated Regulation of Inflammation

Immune cells function as central mediators of the inflammatory cascade in CRS, with their dynamic polarization, tissue infiltration, and bidirectional communication with stromal cells critically shaping the trajectory of inflammation and fibrosis. The reciprocal dysfunction between the heart and kidneys establishes a pro-inflammatory milieu enriched with DAMPs, ROS, and uremic toxins, which collectively orchestrate the recruitment and activation of heterogeneous immune populations, including macrophages, neutrophils, T lymphocytes, and dendritic cells (DCs)-thereby fueling a self-perpetuating cycle of organ injury [84].

Macrophages constitute the predominant immune infiltrate in cardiorenal tissues, exhibiting marked phenotypic plasticity through transitions between pro-inflammatory M1 and anti-inflammatory/pro-fibrotic M2 states [17]. The M1-to-M2 transition is regulated by multiple signaling pathways, including NF-κB, STAT3, and hypoxia-inducible factors, which respond to the local cytokine milieu [85]. In CRS, DAMPs and pro-inflammatory cytokines drive early M1 polarization, contributing to tissue injury, while persistent inflammation promotes a shift toward M2 polarization, which subsequently facilitates the expression of fibronectin, α-smooth muscle actin (α-SMA), and type I collagen, thereby contributing to renal fibrosis [22].

Neutrophils, as first responders to tissue injury, are well-established mediators of inflammation and tissue damage, functioning largely through the release of neutrophil extracellular traps (NETs) [86]. NETs are web-like structures composed of DNA, histones, and granule proteins that trap pathogens but also induce oxidative stress, disrupt endothelial integrity, and activate the NLRP3 inflammasome in macrophages and renal tubular cells, culminating in pyroptosis and IL-1β release [87]. Although direct evidence in human CRS remains limited, preclinical models demonstrate that NET inhibition via DNase I or PAD4 blockade attenuates inflammatory infiltration, oxidative damage, and fibrosis, underscoring the promise of neutrophils as emerging therapeutic targets [88].

T lymphocytes exert divergent regulatory effects on CRS progression through subset-specific functions. The balance between pro-inflammatory T helper (Th) subsets and regulatory T cells (Tregs) is critical in determining the outcome of tissue inflammation. In the context of cardiorenal injury, Th1 cells secrete interferon-γ (IFN-γ) to sustain M1 activation, whereas Th2 cells produce IL-4/IL-13 to drive M2 differentiation and fibrosis [89,90]. Th17 cells further amplify injury via IL-17A–mediated neutrophil recruitment and TGF-β induction [91]. In contrast, regulatory T cells mitigate inflammation through IL-10 and TGF-β secretion, and their depletion in experimental models exacerbates cardiorenal damage [92].

Cross-talk between immune and stromal cells further amplifies the inflammatory-fibrotic axis. Macrophage-derived TGF-β1 and platelet-derived growth factor (PDGF) activate fibroblasts into extracellular matrix (ECM)-producing myofibroblasts, while activated fibroblasts reciprocally secrete C-C motif chemokine ligand 2 (CCL2) and C-X-C motif chemokine ligand 10 (CXCL10) to recruit additional immune cells [93,94]. Endothelial cells, stimulated by ROS and cytokines, upregulate intercellular cell adhesion molecule-1/vascular cell adhesion molecule-1 (ICAM-1/VCAM-1) to facilitate leukocyte adhesion and transmigration, exacerbating vascular dysfunction [95].

The balance between pro- and anti-inflammatory immune responses ultimately dictates the pace of cardiorenal injury and fibrosis in CRS. Therapeutic strategies targeting macrophage polarization show promise in preclinical research, including hypoxia-inducible factor-1α (HIF-1α) inhibition to suppress M1 activation [85]. This process was mainly mediated by PINK1/Parkin-mediated mitophagy, which is consistent with the role of hydrogen sulfide donors in enhancing M1-to-M2 reprogramming [96], and the modulation of the amyloid beta precursor protein (APP)-CD74 axis in endothelial-macrophage communication [97]. Similarly, interventions aimed at the AlkB Homolog 5 (ALKBH5)/Resistin-like alpha (Retnla) [98], Twist1/galectin-3 [99], or *miR-382*/signal regulatory protein-α (SIRP-α)/STAT3 [100] pathways offer novel avenues to disrupt fibrogenic macrophage programming.

Despite these advances, the functional plasticity and context-dependent roles of immune cells necessitate precision approaches that target specific subsets or mediators at defined disease stages. Future research must prioritize elucidating the spatiotemporal dynamics of immune infiltration in CRS, identifying cell-specific biomarkers, and developing combinatorial regimens that concurrently modulate immune responses and fibrotic signaling to halt the progression of this lethal syndrome.

## 4. Fibrosis as the Final Common Pathway

### 4.1 Myofibroblast Activation and Differentiation

While fibrosis is a common endpoint in all CRS subtypes, its temporal progression and underlying drivers differ. In acute subtypes (Type 1 and Type 3), fibrosis may develop rapidly as a maladaptive repair response to acute injury. In chronic subtypes (Type 2 and Type 4), fibrosis progresses insidiously over time, driven by persistent low-grade inflammation and oxidative stress [17]. Type 5 CRS, depending on the underlying systemic disease, may exhibit mixed features. For example, in systemic sclerosis—a prototypical cause of Type 5 CRS—studies have utilized markers like left ventricular mass to characterize this heterogeneity [101].

Fibrosis represents the excessive accumulation of ECM components owing to loss of an appropriate homeostatic balance between ECM synthesis and degradation [102]. The activation of myofibroblasts is a central event in the fibrotic process, with these cells serving as the primary producers of collagen and other ECM proteins [103,104,105]. Myofibroblasts originate from multiple sources. In addition to resident fibroblasts, a substantial proportion derive from epithelial and endothelial cells through epithelial-mesenchymal transition (EMT) and endothelial-mesenchymal transition (EndoMT). This transdifferentiation is primarily driven by pro-fibrotic cytokines, particularly TGF-β1, whose signaling is sustained by the inflammatory and oxidative stress milieu characteristic of CRS [106,107]. Combined with the inflammatory signaling pathway mentioned earlier, its activation process mainly relies on the synergistic regulation of NF-κB, the NLRP3 inflammasome, and HMGB1-related signaling pathways, while being driven by oxidative stress and pro-fibrotic cytokines. Specifically, NLRP3 inflammasome activation releases pro-inflammatory factors such as IL-1β, while HMGB1/RAGE/TLR/NF-κB axis activation produces TNF-α and IL-6, which can directly stimulate resident fibroblasts to activate and differentiate into myofibroblasts. Upstream oxidative stress can indirectly amplify pro-fibrotic signals and accelerate myofibroblast differentiation by activating the TXNIP/NLRP3 pathway and triggering HMGB1 release. In addition, TGF-β, as a core pro-fibrotic cytokine, can be directly upregulated by the NF-κB pathway, thereby promoting sustained activation and collagen secretion of myofibroblasts through the Smad signaling pathway, forming a positive feedback loop consisting of inflammation, oxidative stress, and TGF-β-driven myofibroblast activation [108].

Myofibroblasts, the key effector cells in tissue fibrosis, are mainly derived from resident tissue fibroblasts, with potential contributions from pericytes and fibrocyte progenitors, among other cellular sources [109,110]. In CRS, the reparative process is unique in that it involves a regenerative phase characterized by proliferation and polyploidization, followed by a subsequent pathogenic phase of fibrosis, which is distinct from that observed in other organs [111].

In the fibrotic cardiorenal process of CRS, the number of active myofibroblasts significantly increases, and extracellular matrix proteins are deposited in a degradation-resistant manner. In the kidney, this is characterized by accumulation of collagen-I, -III, and -IV, contributing to glomerulosclerosis and tubulointerstitial fibrosis. In the heart, collagen-I and -III deposition leads to interstitial and perivascular fibrosis, impairing myocardial compliance and function [17,112]. ROS can directly induce inflammation or programmed cell death, which in turn activates fibroblasts to produce ECM proteins [113]. Persistent tissue injury drives an inappropriate pro-fibrotic response, establishing a self-amplifying cycle of tissue damage and repair.

### 4.2 Extracellular Matrix Remodeling

The ECM undergoes significant remodeling during fibrogenesis, with alterations in both composition and structure. ECM accumulation occurs along with the upregulation of matrix metalloproteinases (MMPs) such as MMP-9, which has a wide range of substrates, including ECM components and inflammatory cytokines, implicating MMP-9 in a variety of pathological and age-related processes. There is strong evidence that inflammatory cell-derived MMP-9 exacerbates cardiorenal aging [114]. Tissue inhibitor of metalloproteinases (TIMPs) play crucial roles in regulating ECM turnover by inhibiting MMP activity [115].

In aging hearts and kidneys, there is an imbalance between the natriuretic peptide system (NPS) and RAAS activity, as well as a decline in the degradative pathway, favoring collagen accumulation [20]. The age-mediated alterations in these fibrotic regulator and mediator profiles favor collagen accumulation due to an imbalance between synthesis and degradation [114]. This imbalance is characterized by lower circulating atrial and C-type natriuretic peptides and higher angiotensin II and aldosterone levels in aged rats. Moreover, in aged rats, researchers observed a decrease in collagen-I and -III mRNA levels, accompanied by an increase in *Timp1* mRNA levels, in both aged heart and kidney tissues, while *Tgfb1* expression increased and *Mmp9* decreased specifically in the aged kidney [20].

### 4.3 Intervention Targets for Fibrosis

The TGF-β1/Smad signaling axis has been identified as a master regulator of fibrogenesis, orchestrating fibroblast activation, EMT, and ECM deposition in both cardiac and renal tissues [116,117]. Notably, fibroblast-specific deletion of *Smad3* significantly attenuates pressure overload-induced cardiac fibrosis, underscoring the cell-type-specific importance of this pathway in tissue remodeling [118]. In the kidney, TGF-β1/*Smad3* activation similarly drives tubulointerstitial fibrosis by stimulating EMT and ECM accumulation, with *Smad3* acting as a critical transcriptional hub that integrates signals from angiotensin II, advanced glycation end products, and inflammatory mediators [119]. Emerging evidence highlights the pivotal role of non-coding RNAs as epigenetic amplifiers of TGF-β-driven fibrosis. MicroRNAs such as *miR-21* form feed-forward loops that potentiate TGF-β signaling and promote fibrogenesis, whereas the *miR-29* and *miR-200* families exert anti-fibrotic effects by inhibiting ECM deposition and EMT, respectively [120]. Long non-coding RNAs (lncRNAs) further modulate fibrotic responses by regulating chromatin architecture, mRNA stability, and protein scaffolding within the TGF-β/Smad network [121]. In cardiac fibrosis, ncRNAs, including miRNAs, lncRNAs, and circRNAs, form intricate regulatory circuits that fine-tune fibroblast activation and ECM dynamics, with dysregulation contributing to adverse remodeling in atrial fibrillation and post-infarction settings [122]. Similarly, in renal fibrosis, specific lncRNAs modulate the TGF-β/Smad and Wnt/β-catenin pathways, thereby influencing disease progression in diabetic nephropathy and chronic allograft rejection [123,124,125]. This ncRNA-mediated amplification of fibrotic signaling not only accelerates tissue scarring but also impairs regenerative capacity, highlighting these molecules as promising diagnostic biomarkers and therapeutic targets for CRS-related fibrosis.

Targeting myofibroblasts is an important therapeutic direction because, as α-SMA-positive cells, they are the main source of pathological ECM synthesis [109,126]. Restoring ECM homeostasis in fibrotic disorders necessitates precise rebalancing of the activity of MMPs and their endogenous inhibitors, the TIMPs. In fibrotic microenvironments, TIMP overexpression, particularly that of TIMP-1, frequently suppresses MMP-mediated degradation, leading to the pathological accumulation of structural collagens, including types I, III, and IV [127]. This disproportion is patterned in a variety of organ systems: in interstitial lung disease, the decreased MMP-9/TIMP-1 ratio in bronchoalveolar lavage fluid is correlated to a high degree with extensive fibrosis and a high amount of collagen deposition [128]. In the context of liver cirrhosis, activated HSCs stimulate excess ECM production, and the upregulation of TIMP suppresses normal matrix turnover [127]. Preclinical evidence robustly demonstrates that the therapeutic modulation of this proteolytic equilibrium can attenuate fibrosis. For instance, interventions that downregulate TIMP-1 or enhance MMP-9 activity have been shown to reduce collagen accumulation in animal models of cardiac, renal, and pulmonary fibrosis [129]. The clinical translation of MMP/TIMP-targeted anti-fibrotic strategies is still severely constrained, even though they have been strongly supported by preclinical evidence. The bidirectional and context-dependent roles of MMP-9, which acts as both a pro-fibrotic and anti-fibrotic mediator depending on disease stage and tissue type, complicate therapeutic targeting [130].

The inflammatory microenvironment drives fibrosis through continuous interactions between immune cells and stromal cells. In the positive feedback loop between inflammation and fibrosis, the release of IL-1β mediated by NLRP3 inflammasome is particularly critical, which is activated under oxidative stress and uremic toxin stimulation to promote fibrogenesis [131]. In addition, the Wnt/β-catenin and TWEAK/Fn14 pathway, as a cross-signal node, can induce fibroblast proliferation and renal tubular injury, in addition to inhibiting the expression of protective factors such as Klotho [61,132].

**Fig. 1.**
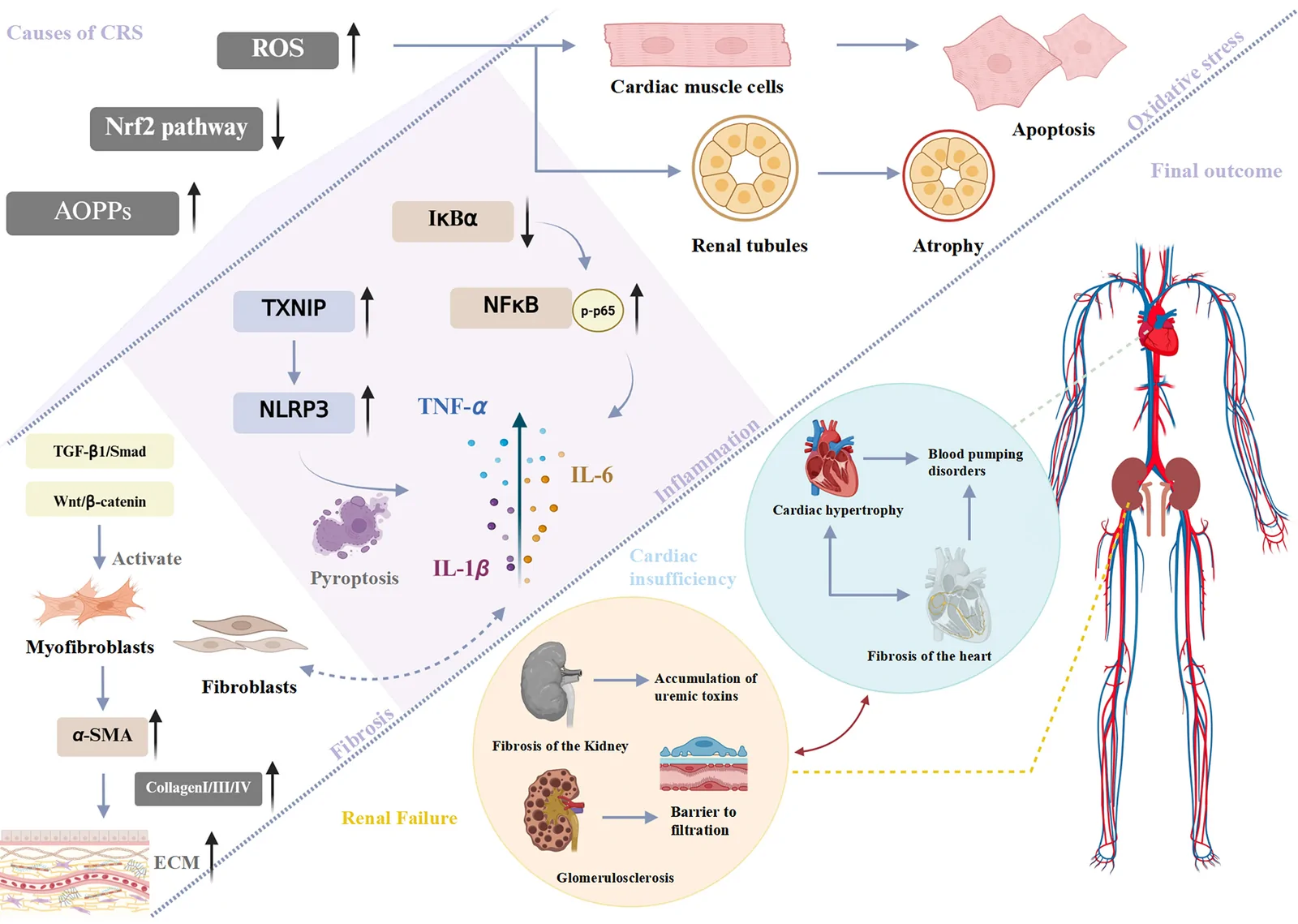
**Schematic diagram of the pathological mechanism of CRS**. Upstream triggers (elevated ROS/AOPPs, impaired Nrf2 signaling) disrupt redox homeostasis. These factors activate a central inflammatory hub via TXNIP-NLRP3 inflammasome (pyroptosis, IL-1β release) and NF-κB (TNF-α, IL-6 transcription) pathways. In the kidney, inflammation drives renal injury and fibrosis (tubular atrophy, glomerulosclerosis, myofibroblast activation, ECM deposition), leading to renal failure and uremic toxin accumulation. In the heart, oxidative stress and inflammation induce cardiac remodeling and dysfunction (cardiomyocyte apoptosis, hypertrophy, interstitial fibrosis), impairing pump function. Bidirectional crosstalk between cardiac and renal dysfunction creates a self-reinforcing vicious cycle, propagating multi-organ injury and progressive CRS deterioration, the upward arrow indicates promotion/activation, while the downward arrow indicates suppression/inhibition. CRS, cardiorenal syndrome; ROS, reactive oxygen species; AOPPs, advanced oxidation protein products; Nrf2, nuclear factor erythroid 2-related factor 2; TXNIP-NLRP3, thioredoxin-interacting protein-NOD-like receptor family pyrin domain-containing protein 3; IL, interleukin; NF-κB, nuclear factor κB; TNF-α, tumor necrosis factor-α; ECM, extracellular matrix.

Targeting these immune-stromal communication axes has emerged as a promising therapeutic strategy to disrupt the inflammatory-fibrotic cycle in CRS. Pharmacological inhibition of the NLRP3 inflammasome with MCC950 attenuates IL-1β release and subsequent fibroblast activation in experimental fibrosis models [133]. Blockade of the TWEAK/Fn14 axis using neutralizing antibodies reduces both cardiac and renal fibrosis in preclinical CRS models [61]. Additionally, inhibitors of the Wnt/β-catenin pathway, such as ICG-001, have shown anti-fibrotic effects in kidney and heart tissues [60]. Collectively, these approaches aim to interrupt the crosstalk between immune cells and fibroblasts, thereby halting the self-perpetuating cycle of inflammation and fibrosis that drives CRS progression (Fig. 1).

## 5. Integrated Therapeutic Strategies for Cardiorenal Syndrome

### 5.1 Overview of Current Therapeutic Approaches

Current therapeutic strategies for CRS increasingly focus on targeting the shared pathophysiological pathways of oxidative stress, inflammation, and fibrosis, rather than addressing cardiac and renal dysfunction in isolation. Among these, sodium-glucose cotransporter 2 (SGLT2) inhibitors have emerged as a cornerstone therapy due to their robust cardiorenal protective effects. In addition to hypoglycemic effects, SGLT2 inhibitors can provide significant cardiorenal protection by improving myocardial energy metabolism, optimizing ventricular load state, promoting diuresis and sodium excretion, and inhibiting pro-fibrotic effects related to inflammation and oxidative stress [134,135,136]. Preclinical studies have further revealed that SGLT2 inhibitors can inhibit Na^+^/H^+^ exchanger 1 (NHE1) activity in myocardial cells and reduce autophagic cell death (autosis), thereby improving ventricular remodeling and cardiac function after myocardial infarction [137]. In addition, empagliflozin has been confirmed to enhance myocardial metabolic efficiency, reduce myocardial fibrosis, and reverse left ventricular hypertrophy in animal models of inherited cardiomyopathy through a mechanism involving mTOR signaling pathway inhibition and substrate metabolic flux normalization [138]. With respect to renal protection, SGLT2 inhibitors reduce the intraglomerular pressure by activating the tubuloglomerular feedback mechanism, and reduce the reabsorption load of albumin by the proximal tubule, thereby alleviating renal tubular lipotoxicity, inflammation, and fibrosis [139]. Dapagliflozin can also reduce oxidative stress and improve left ventricular ejection fraction in animal models of heart failure by changing myocardial substrate utilization patterns, increasing ketone body oxidation, reducing pyruvate oxidation, and enhancing mitochondrial redox status [140]. Proteomics and metabolomics studies provide a new perspective for analyzing the cardiorenal-protective mechanism of SGLT2 inhibitors [141]. Existing evidence shows that its benefits are derived from multi-dimensional regulation of hemodynamics, metabolic homeostasis, the inflammation-fibrosis axis, and organelle function. These findings offer insight into the molecular target network of SGLT2 inhibitors, supporting their use as cornerstone drugs for the treatment of heart failure and CKD.

Natural bioactive compounds exhibit multi-target therapeutic potential in modulating inflammatory and fibrotic pathways relevant to CRS, although their application specifically for CRS remains largely in the preclinical stage. Preclinical studies have demonstrated that Salvianolic acid B, for instance, reduces inflammation and fibrosis by inhibiting the NF-κB signaling pathway in macrophages, thereby attenuating M1 polarization and adipose tissue fibrosis [142]. Similarly, curcumin, specifically its analogue EF24, ameliorates pulmonary fibrosis by preventing alveolar epithelial cell senescence through PTEN activation and suppression of the AKT/mTOR/NF-κB axis, which also inhibits fibroblast activation driven by senescent cells [143]. In the context of CRS, emerging evidence suggests that these agents may exert separate protective effects on the heart and kidneys. For instance, formononetin, an isoflavone compound, has been shown to alleviate CRS type 4 by targeting Hippo-YAP signaling, thereby reducing mitochondrial oxidative stress and preserving cardiac function in CKD-induced cardiomyopathy mouse models [144]. Specifically for renal protection, omega-3 polyunsaturated fatty acids have demonstrated efficacy in alleviating renal fibrosis in chronic kidney disease by reducing macrophage activation and infiltration [145], while curcumin derivatives have demonstrated cardioprotective effects in heart failure models [146]. Despite these promising mechanisms, no single anti-fibrotic therapy has demonstrated clear clinical efficacy in CRS, primarily due to disease heterogeneity and the lack of validated stratification biomarkers. The complexity of CRS limits the effectiveness of monotherapies targeting isolated pathways such as NF-κB or NLRP3 signaling. Experimental evidence suggests that GLP-1 receptor activation attenuates pulmonary fibrosis by disrupting the NLRP3 inflammasome/PFKFB3-driven glycolysis interaction and reducing histone lactylation-mediated pro-fibrotic gene expression, highlighting the centrality of NLRP3 in fibrogenesis across tissues [147]. However, translating these findings to CRS will require accounting for organ-specific microenvironmental differences that may alter inflammasome regulation. Notably, recent studies have identified natural compounds that simultaneously target both organs through shared pathways. For example, the bergamot polyphenolic fraction has been shown to reduce oxidative stress and improve both kidney and heart function in unilateral renal artery ligation rat models, suggesting potential for CRS applications [148]. Future therapeutic strategies should therefore integrate the pleiotropic actions of natural compounds within a precision medicine framework tailored to CRS endotypes. Key priorities include: (1) identifying accessible, endotype-specific biomarkers (e.g., CXCL9/CXCL10 for Type 1 CRS, CCL26 for Type 2 CRS, and CSF3 for Type 3 CRS); (2) repurposing multi-target agents like salvianolic acid B and EF24-curcumin in biomarker-stratified trials; and (3) designing rational combinations that concurrently inhibit NLRP3 activation, epithelial senescence, and macrophage polarization. Given that glucagon-like peptide-1 receptor (GLP-1R) agonism disrupts NLRP3-dependent fibroblast activation and salvianolic acid B suppresses NF-κB driven inflammation, synergistic regimens could break the self-perpetuating cycle of inflammation and fibrosis, ultimately improving long-term outcomes in CRS.

### 5.2 Future Directions and Unmet Needs

Although some research progress has been related to the treatment of CRS, these therapeutic advances have not yet addressed all the key needs and unmet clinical pain points associated with this disease [149]. First, the early-stage diagnosis of this condition may remain insufficient, such that many patients are first diagnosed when the disease is more advanced and less responsive to treatment. Future efforts aimed at the development of more sensitive biomarkers, such as urinary peptidomics, are thus needed to screen high-risk populations [150]. Second, currently available therapies focus primarily on symptom relief rather than fundamental modification of the disease process. Although SGLT2i administration has shown significant benefits in improving cardiac and renal outcomes, including delaying renal function decline and reducing hospitalization rates for heart failure and all-cause mortality, regardless of diabetes status, therapeutic strategies for fibrosis regression and cell regeneration remain exploratory [151].

Underrepresentation in clinical trials is another persistent challenge. Patients with CRS are often excluded from major trials, resulting in treatment recommendations that are mostly derived from subgroup analyses [152]. It is worth noting that the multidisciplinary collaboration model has been shown to significantly improve the prognosis of CRS patients. A comprehensive management plan integrating cardiology, nephrology, pharmacy, and nursing teams can optimize drug therapy, in addition to improving the efficiency of diuretic use through accurate volume assessment [153]. Moreover, the discovery of emerging functions related to metabolites derived from the gut microbiota, such as trimethylamine-N-oxide (TMAO), offers a new perspective for understanding the systemic pathophysiology of CRS, and may give rise to intervention strategies targeting the microbiome [154].

**Fig. 2.**
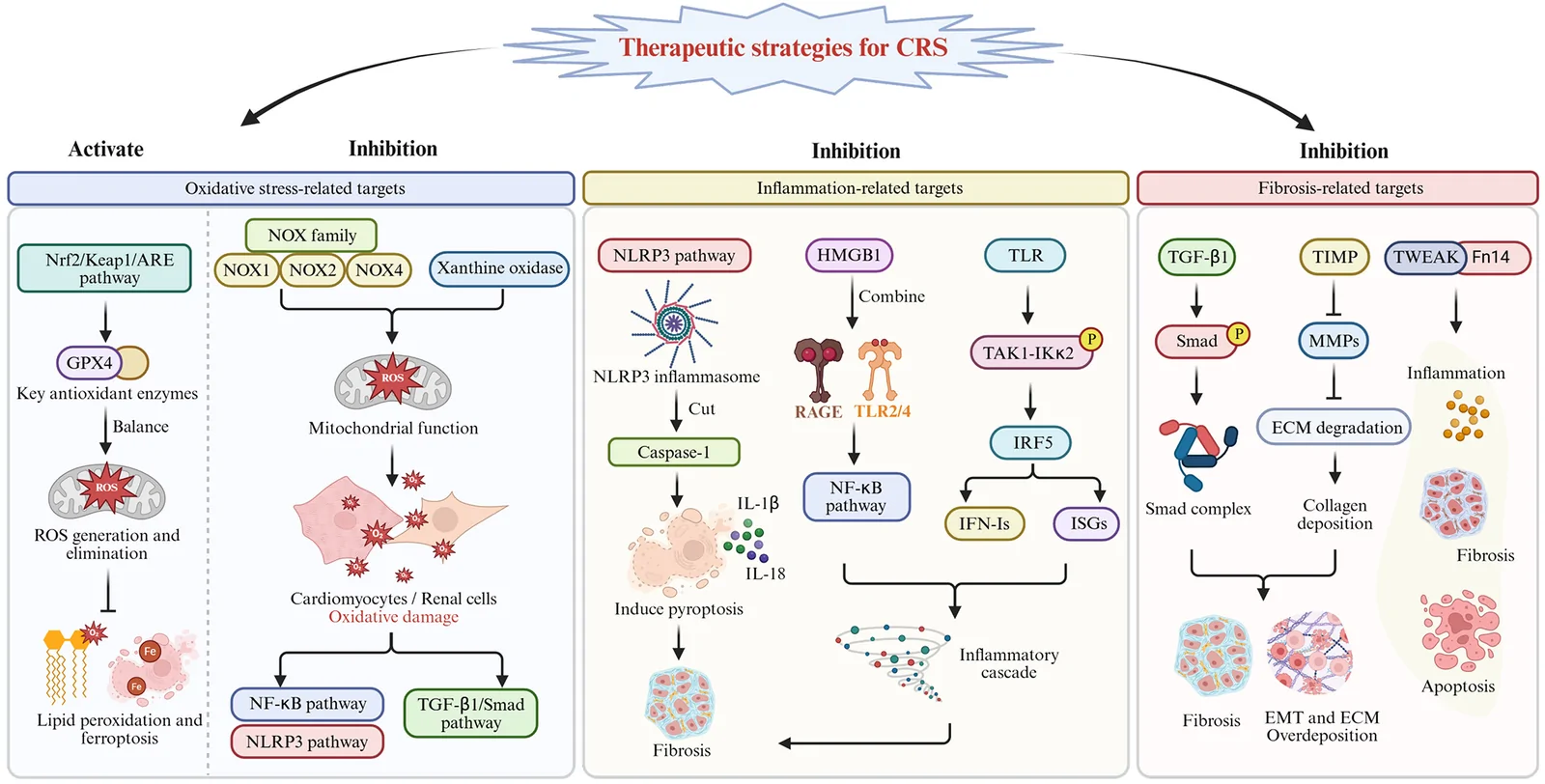
**Therapeutic strategies for CRS targeting oxidative stress, inflammation, and fibrosis**. Oxidative stress-related targets (left panel): Activation of the Nrf2/Keap1/ARE pathway upregulates key antioxidant enzymes (GPX4), restoring redox balance, inhibiting ROS overproduction, and alleviating lipid peroxidation and ferroptosis. Inhibition of NOX family members (NOX1, NOX2, NOX4) and xanthine oxidase preserves mitochondrial function, reduces oxidative damage to cardiomyocytes and renal cells, and suppresses downstream pro-inflammatory (NF-κB, NLRP3) and pro-fibrotic (TGF-β/Smad) signaling. Inflammation-related targets (middle panel): Inhibition of the NLRP3 inflammasome attenuates caspase-1 activation, IL-1β/IL-18 secretion, and pyroptosis. Blocking HMGB1 interaction with RAGE/TLR2/4 disrupts NF-κB pathway activation. Inhibition of the TLR-TAK1-IKKβ axis suppresses IRF5-mediated proinflammatory cytokine production, thereby dampening the inflammatory cascade and subsequent fibrosis. Fibrosis-related targets (right panel): Inhibition of the TGF-β1-Smad pathway reduces Smad complex formation and collagen deposition. Inhibiting TIMP activity enhances MMP-mediated ECM degradation, while targeting the TWEAK axis mitigates inflammation, fibrosis, and apoptosis, ultimately ameliorating EMT and ECM accumulation.

Further studies should involve CRS-specific trials with more diversified patient groups, such as older and/or multimorbid patients. Moreover, there is no available long-term safety and benefit information, particularly in the case of new treatments like senolytics [155,156]. Interdisciplinary collaboration will be central to moving the field forward. Collaboration among cardiorenal scientists, basic researchers, bioinformaticians, and industry will accelerate mechanistic discovery and therapeutic innovation [157]. At the same time, patient education and self-management programs are increasingly important to enhance treatment benefits by improving compliance and lifestyle. Together, these efforts will improve the prognosis of CRS (Fig. 2).

## 6. Conclusions and Prospects

The future of CRS diagnosis and treatment hinges on a paradigm shift toward mechanism-oriented, integrated multi-target precision interventions. To achieve this, three key priorities must be addressed. Deepening the understanding of the core oxidative stress–inflammation–fibrosis axis is essential to identify nodal targets and establish a theoretical basis for disease stratification. In addition, therapeutic strategies must leverage the pleiotropic benefits of existing agents while developing rational combination regimens. Advancing translational research will require addressing unmet needs in early diagnosis and clinical trial design. Moreover, inclusive clinical trials involving older adults, patients with comorbidities, and different CRS subtypes will validate the efficacy and safety of novel combination therapies.

In summary, CRS represents a quintessential example of a multi-organ disease driven by interconnected pathological pathways, demanding a holistic approach that transcends isolated target inhibition. By integrating insights from basic science and clinical practice, refining precision medicine frameworks, and advancing synergistic combination therapies, we can disrupt the vicious cycle of cardiorenal dysfunction, improve long-term patient outcomes, and ultimately reduce the substantial public health burden of CRS.
